# The molecular basis of flavonoid biosynthesis response to water, light, and temperature in grape berries

**DOI:** 10.3389/fpls.2024.1441893

**Published:** 2024-08-27

**Authors:** Tianci Shi, Yue Su, Yibin Lan, Changqing Duan, Keji Yu

**Affiliations:** ^1^ Center for Viticulture and Enology, College of Food Science and Nutritional Engineering, China Agricultural University, Beijing, China; ^2^ Key Laboratory of Viticulture and Enology, Ministry of Agriculture and Rural Affairs, Beijing, China

**Keywords:** proanthocyanidin, anthocyanin, flavonoid, water, light, temperature, phytohormones, grape

## Abstract

Flavonoids, including proanthocyanidins (PAs), anthocyanins and flavonols are essential secondary metabolites that contribute to the nutritional value and sensory quality of grape berry and red wine. Advances in molecular biology technology have led to substantial progress in understanding the regulation of flavonoid biosynthesis. The influence of terroir on grape berries and wine has garnered increasing attention, yet its comprehensive regulatory network remains underexplored. In terms of application, environmental factors such as water, light, and temperature are more easily regulated in grapevines compared to soil conditions. Therefore, we summarize their effects on flavonoid content and composition, constructing a network that links environmental factors, hormones, and metabolites to provide a deeper understanding of the underlying mechanisms. This review enriches the knowledge of the regulatory network mechanisms governing flavonoid responses to environmental factors in grapes.

## 1 Introduction

Grapevine (*Vitis vinifera*) is one of the most widely cultivated and consumed fruits in the world, and its use in wine production is attracting more interest due to its economic and health benefits (Beres et al., 2017; Wei et al., 2023). Proanthocyanidins (PAs, polymers of flavan-3-ols), anthocyanins and flavonols are abundant flavonoids in grapes. These compounds not only protect grapes from UV rays, pests and diseases, but also determine the color, astringency, and stability of red wine (Winkel-Shirley, 2002; Flamini et al., 2013). Flavonoids, characterized by a C6-C3-C6 backbone, are classified into anthocyanins, flavonols, and flavan-3-ols in grape depending on the oxidation and substitution state of the pyran ring in the carbon skeleton (Ferreyra et al., 2012; Gouot et al., 2019a). Anthocyanins are primarily located in the skin of grape, accumulating significantly at the veraison stage and peaking around the harvest stage. Flavonols are also mainly distributed in the skin, with their synthesis starting at the flowering stage, slowing down in the early stages of fruit development, and resuming during fruit ripening. Flavan-3-ols are detected in both the grape skin and seeds, and their synthesis occurs from anthesis until the onset of ripening (Downey et al., 2004; Bogs et al., 2005; He et al., 2010).

**Figure 1:**
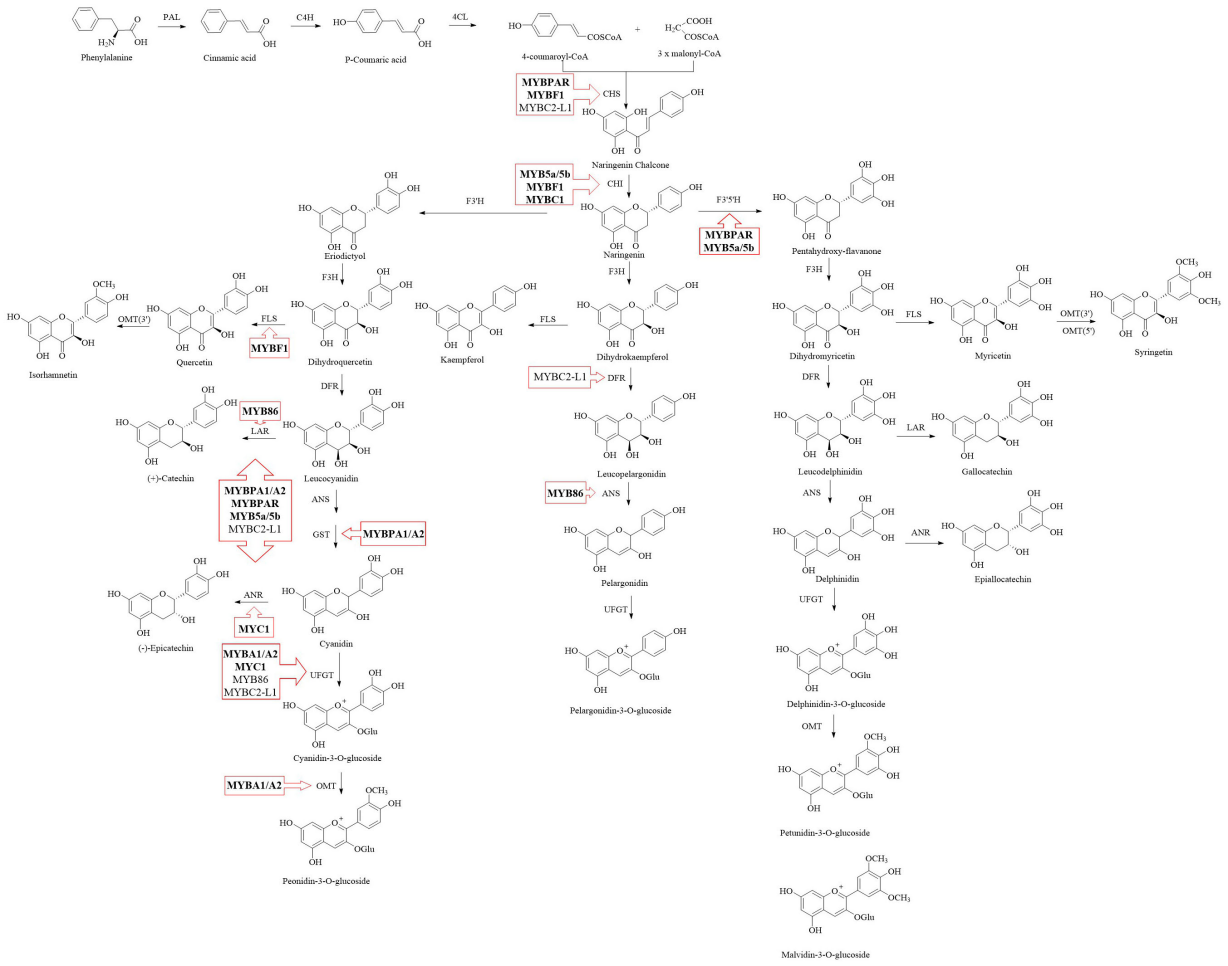
The metabolic pathway of flavonoid in grape. Positive regulators are indicated in bold text, while negative regulators are shown in normal text. PAL, phenylalanine ammonia-lyase; C4H, cinnamate-4-hydroxylase; 4CL, 4-coumarate, CoA-ligase; CHS, chalcone synthase; CHI, chalcone isomerase; F3H, flavanone 3-hydroxylase; F3’H, flavanone 3’-hydroxylase; F3’5’H, flavanone 3’5’-hydroxylase; DFR, dihydroflavonol 4-reductase; ANR, anthocyanidin reductase; ANS, anthocyanidin synthase; FLS, flavonol synthase; UFGT, UDP-glucose, flavonoid 3-*O*-glucosyltransferase; OMT, *O*-methyltransferase.

The flavonoid pathway in grapevine, derived from the shikimate and phenylpropanoid pathways, has been well-characterized (Ferreira et al., 2018; Gouot et al., 2019a). As shown in **Figure 1**, phenylalanine, a product of shikimate pathway, is catalyzed by a series of enzymes, including phenylalanine ammonia-lyase (PAL), cinnamate 4-hydroxylase (C4H) and 4-coumarate coenzyme A ligase (4CL), to form 4-coumaroyl-CoA, a precursor in the flavonoid pathway. Subsequently, 4-coumaroyl-CoA and malonyl-CoA are catalyzed by chalcone synthase (CHS) and chalcone isomerase (CHI) to form chalcone, a 15-carbon flavonoid skeleton, and then naringenin, an intermediate product of flavonoid metabolism, which enters various flavonoid metabolic branches (Holton and Cornish, 1995). With the catalysis of flavanone hydroxylases (F3H, F3’H or F3’5’H), naringenin is converted to dihydroflavonols including dihydrokaempferol, dihydromyricetin and dihydroquercetin (Bogs et al., 2006). The next stage involves branching: one branch produces flavonols from dihydrokaempferol by flavonol synthase (FLS), while the other, controlled by dihydroflavonol 4-reductase (DFR), converts dihydromyricetin and dihydroquercetin into leucodelphinidin and leucocyanidin (possessing 2,3-*trans* conformation), respectively (Martens et al., 2002). Leucodelphinidin and leucocyanidin are then converted to delphinidin and cyanidin via the combined activities of anthocyanidin synthase (ANS) and glutathione-*S*-transferase (GST) (Eichenberger et al., 2023). They also can be catalyzed by leucoanthocyanidin reductase (LAR) producing 2,3-*trans* flavan-3-ol (e.g. (+)-catechin) (Yu et al., 2019). Additionally, the delphinidin and cyanidin are converted to 2,3-*cis* flavan-3-ol (e.g. (-)-epicatechin) with the catalysis of anthocyanidin reductase (ANR) (Xie et al., 2003; Bogs et al., 2005; Jun et al., 2021; Yu et al., 2022). These flavan-3-ol monomers participate in the condensation process of PA as starter units (Dixon et al., 2005; Yu et al., 2023). Flavan-3-ol carbocations, derived either from 2,3-*trans*-leucoanthocyanidins or from 2,3-*cis*-leucoanthocyanidins produced by ANR, serve as the direct extension units that form C4-C8 or C4-C6 bonds with the upper unit in non-enzymatic PA polymerization (Wang et al., 2020a; Jun et al., 2018, Jun et al., 2021; Yu et al., 2022, Yu et al., 2023). For another branch, anthocyanidins can further undergo modifications such as glycosylation, acylation, and methylation mediated by UDP-glucose (UFGT), acyltransferases and *O*-methyltransferase (OMT) respectively, to produce anthocyanins (He et al., 2008). Finally, flavonoids synthesized on the endoplasmic reticulum are typically transported by transporter proteins, including GST and multidrug and toxic extrusion transporter (MATE), to vesicles for storage (Castellarin et al., 2007a).

It is believed that flavonoid synthesis is mainly controlled by the MYB-bHLH-WD40 (MBW) complex, with the MYB protein playing a central role (Xu et al., 2015). Among the R2R3-MYB transcription factors, several of them have been identified in grapevine for their roles in flavonoid metabolism. *VvMYB5a*, expressed in the early stages of berry development, and *VvMYB5b*, expressed throughout berry development, activate the promoters of upstream flavonoid pathway genes (*VvCHI*, *VvF3’5’H*) and the PA biosynthesis pathway-related structural genes including *VvLAR1* and *VvANR* (Deluc et al., 2006, Deluc et al., 2008). Overexpression of *VvMYB5a* and *VvMYB5b* in tobacco (*Nicotiana tabacum*) leads to high accumulation of anthocyanins and PAs via upregulating the expression of genes involved in the flavonoid biosynthetic pathway, such as *VvCHS*, *VvCHI*, *VvF3H*, and *VvDFR*. Moreover, *VvCHS* and *VvCHI* can also be regulated by VvMYBF1, but VvMYBF1 mainly regulates *VvFLS* to produce flavonols, which compete with PAs and anthocyanins synthesis (Czemmel et al., 2009). VvMYBA1 and VvMYBA2 are the key positive regulators of anthocyanin synthesis in grape berries, promoting the expression of *VvUFGT*, *VvGST*, *VvOMT* and *Vv3AT* genes at the pre-veraison. stage (Kobayashi et al., 2004; Cutanda-Perez et al., 2009; Rinaldo et al., 2015). Matus et al. (2017) reported that VvMYBA1, VvMYBA6.1 and VvMYBA7 regulate the synthesis of anthocyanins in young leaves and tendrils of ‘Pinot Noir’ and buds of ‘Corvina Veronese’ by activating the promoter of *VvUFGT*, *VvOMT* and *VvF3’5’H* genes, with only VvMYBA1 inducing *VvF3’5’H*. VvMYBPA1 and VvMYBPA2, mainly expressed in seeds and skin in grapevine respectively, result in the accumulation of PAs by significantly upregulating transcript levels of *VvANR* and *VvLAR1* (Bogs et al., 2006; Terrier et al., 2009). Koyama et al. (2014) suggested that VvMYBPAR also regulate PAs content via controlling *VvLAR2* expression. Additionally, there are transcription repressors involved in flavonoid synthesis. VvMYBC2-L1/2/3 negatively regulate anthocyanin biosynthesis by inhibiting the expression of *VvCHI*, *VvCHS*, *VvDFR*, and *VvUFGT* (Cavallini et al., 2015; Zhu et al., 2019). VvMYBC2-L1 also down-regulates *VvMYBPA1*, *VvMYBPA2*, *VvDFR*, *VvLDOX*, *VvANR*, *VvLAR1* and *VvLAR2* expression to suppress the PAs synthesis (Huang et al., 2014). Cheng et al. (2021) found VvMYB86 promotes PAs synthesis through the up-regulation of *VvLAR* transcription and inhibits anthocyanin biosynthesis via the down-regulation of *VvANS* and *VvUFGT* expression. Among bHLH regulators, it has been confirmed that VvMYC1 can interact with VvMYB5a, VvMYB5b, VvMYBA1/A2, and VvMYBPA1, resulting in the induction of the promoter activities of *VvUFGT*, *VvANR*, and *VvCHI* (Hichri et al., 2010). The studies from Matus et al. (2010) and Jiu et al. (2021) reported that WDR1 enhances the anthocyanin accumulation, by forming the complex with VvMYBA2 and VvMYCA1.

The accumulation of flavonoid is affected by both macro- and micro-climates in vineyard (Downey et al., 2006; Gutierrez-Gamboa et al., 2017; Martinez-Gil et al., 2018). In a specific vineyard, environmental factors such as water, light, and temperature can be adjusted through viticulture practices to regulate the qualities of grape berries and wines (Mori et al., 2007a; Azuma et al., 2012; Rienth et al., 2021). A comprehensive understanding of the mechanisms that regulate flavonoid accumulation in response to environmental factors can contribute to the precise control of fruit traits through cultivation. The enzymes and transcription factors responsible for the biosynthesis of anthocyanins and PAs in grapevine have been extensively studied in the context of spatio-temporal accumulation of metabolites, providing the basis for interpreting how gene expression patterns cause grape berry traits under various environmental conditions. However, the lack of suitable working models to understand conflicting results from field experiment reflects the fact that the interaction between genetic background of grapevine and environmental factors has not been fully resolved. In terms of the remodeling of flavonoid biosynthesis network under various abiotic stresses, the essential roles of phytohormones (e.g. abscisic acid (ABA), ethylene, melatonin, gibberellin GA, brassinolide(BR)) in mediating the accumulation of anthocyanins and PAs in grapevine has been revealed by metabolomics and transcriptomic studies in recent years (Mori et al., 2005a; Loreti et al., 2008; Sun et al., 2017).

## 2 The ABA-centered flavonoid biosynthesis regulation network under water deficit

**Figure 2:**
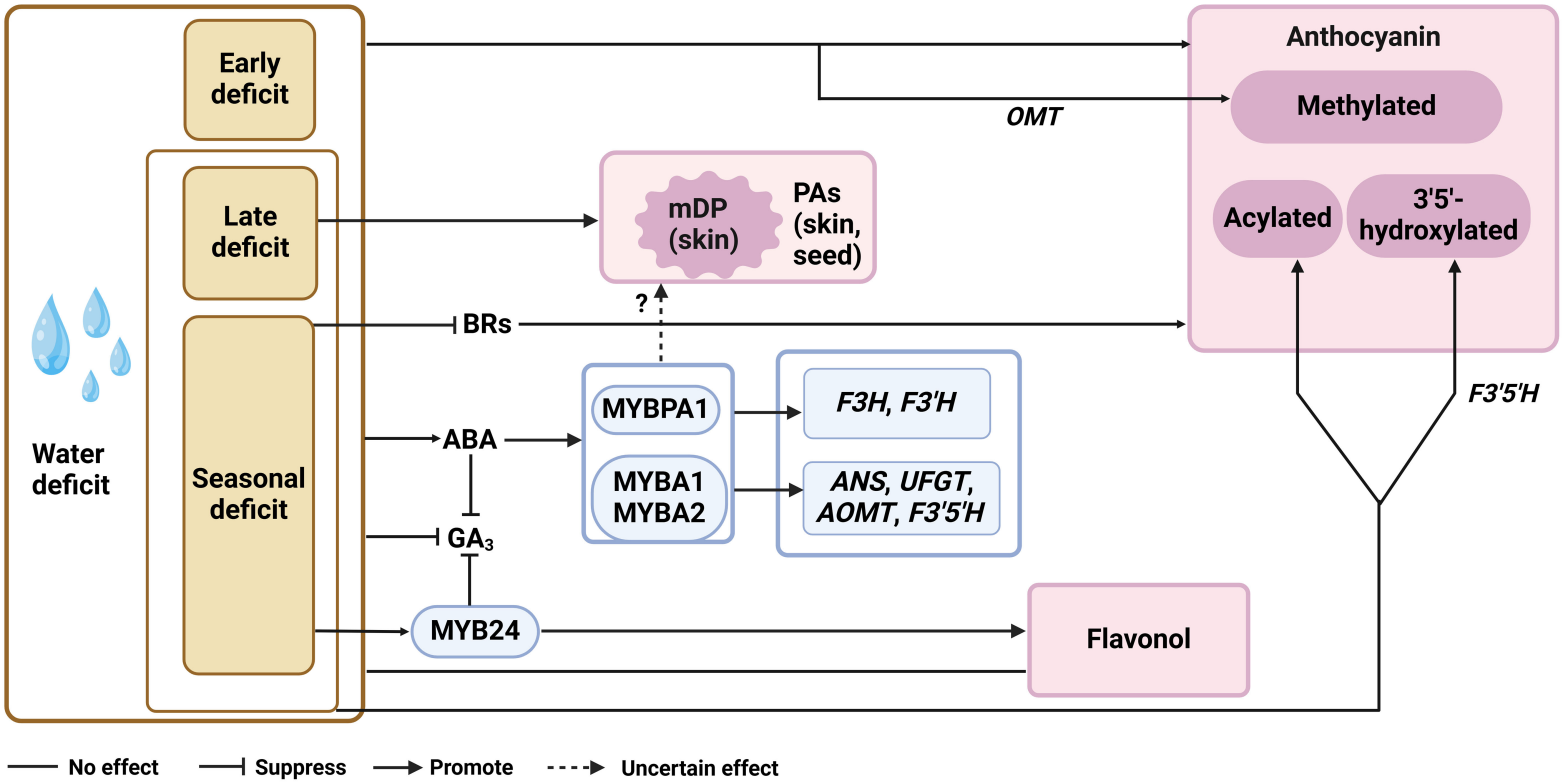
The ABA-centered flavonoid regulation network under water deficit. mDP, mean polymerization degrees; ABA, abscisic acid; GA_3_, gibberellin, BRs, brassinolide; F3H, flavanone 3-hydroxylase; F3’H, flavanone 3’-hydroxylase; F3’5’H, flavanone 3’5’-hydroxylase; ANS, anthocyanidin synthase; UFGT, UDP-glucose, flavonoid 3-*O*-glucosyltransferase; OMT, *O*-methyltransferase.

The current understandings of the effects of water condition on berry qualities are mainly from the practice of regulated deficit irrigation (RDI), a strategy to balance the water usage and the yield (Costa et al., 2007). The modulation of flavonoid biosynthesis in grape berry under water deficit is a complex process, as it may be affected by at least macro climate, grape variety, rootstock, drought timing and even berry size (Roby and Matthews, 2004; Kuhn et al., 2014; Gambetta et al., 2020; Afifi et al., 2021). Moreover, the parameter for representing the water deficit extent varies among studies, such as evapotranspiration (usually ranged from 30% to 80%) and water potentials of leaves or stems (usually ranged from -1.4 to -0.6). It is therefore plausible to observe conflict conclusions drawn from different experiments. By revisiting the existing studies, we here summarized the general molecular mechanism regarding the response of PA and anthocyanin pathways to water deficit, and proposed the omitted aspects that hinder answering the lingering questions from various studies. The commonly used RDI strategies in field studies include the early deficit (from berry setting to veraison), the late deficit (from verasion to harvest) and the seasonal deficit (from berry setting to harvest). It has repeatedly been shown that water deficit has no effect on flavonols, although increases anthocyanin level in red grape skins at harvest, regardless the timing of RDI (Castellarin et al., 2007a; Castellarin et al., 2007b; Deluc et al., 2009; Caceres-Mella et al., 2017; Yang et al., 2020a). This is in line with the increased transcription levels of *VvPAL*, *VvCHS*, *VvF3H*, *VvF3’H*, *VvDFR*, *VvANS*, *VvGST* and *VvUFGT* responsible for anthocyanin biosynthesis in the corresponding RDI treatments (Castellarin et al., 2007a; Deluc et al., 2009; Yang et al., 2020a) (**Figure 2**). All three RDI strategies enhance the methylation of anthocyanins, while the late and seasonal RDI but not the early water deficit significantly increases the levels of delphindin-based and acylated anthocyanins in grape berries (Castellarin et al., 2007a; Olle et al., 2011; Yang et al., 2020a). This suggests that *VvOMT* is essentially responsive to water deficit across the berry development, while *VvF3’5’H* and *Vv3AT* are mainly sensitive to water status post-veraison. However, the patterns of the detected *VvF3’5’H* transcript levels are not always well-correlated with delphindin derivates accumulations among different RDI treatments (Castellarin et al., 2007a). It is noteworthy that *VvF3’5’H* gene family in grapevine possess more than ten isoforms, and the levels of 3’5’-OH anthocyanins are dependent on the abundance of *VvF3’5’H* transcripts pool in grapes (Falginella et al., 2010). Thus, it will be more informative to include the expression data of multiple *VvF3’5’H* gene isoforms when aiming at clarifying the mechanism about the response of anthocyanin hydroxylation extent to water deficit timing. Under both early and late water deficit treatments, the enhanced anthocyanin accumulation in grape berry associates with ripening acceleration, the process mainly regulated by ABA (Castellarin et al., 2007a). Transcriptome and metabolite data both showed that seasonal water deficit can promote ABA synthesis in red grape berries, especially at veraison (Deluc et al., 2009). And the effect of seasonal RDI on anthocyanin accumulation can be diminished by applying ABA synthesis inhibitor nordihydroguaiaretic acid (NDGA) (Guo et al., 2021). These findings suggest the pivotal role of ABA to enhance anthocyanin biosynthesis in grape berries under water deficit stress. The exogenous ABA application studies showed that most of flavonoid genes activated by ABA in grape berries are overlapped with that triggered by water deficit, including three flavonoid activators VvMYBA1, VvMYBA2 and VvMYBPA1 (Koyama et al., 2010; Caceres-Mella et al., 2017; Sun et al., 2019; Guo et al., 2021). Genetic evidence showed that VvMYBA1/2 target to genes in the entire anthocyanin pathway except *VvF3H* and *VvF3’H*, while VvMYBPA1 activates *VvF3H*, *VvF3’H* and two PA branch genes *VvLAR*1 and *VvANR* (Terrier et al., 2009; Rinaldo et al., 2015). Thus, it is likely that VvMYBA1/2 and VvMYBPA1 complement with each other for channeling the flux to anthocyanins regulated by ABA in grape berries under water deficit stress (**Figure 2**).

Although *VvMYBPA1* is crucial for PA accumulation in the context of grape berry development (Bogs et al., 2007), its enhanced expression seems not effectively promote PA biosynthesis under water deficit stress. Numerous studies have shown that water deficit has little effect on PA accumulation in grape berries at harvest, or its effect on grape PA content does not show a stable trend among different years (Castellarin et al., 2007a; Bucchetti et al., 2011; Olle et al., 2011; Genebra et al., 2014; Herrera et al., 2015; Yu et al., 2016; Caceres-Mella et al., 2017). Compositional analysis suggests that it is the mDP of PA, but not the gallyollation extent of PA, increases in grape skins under long term or late water deficit (Caceres-Mella et al., 2017) (**Figure 2**). At the transcription level, *VvANR* expression in grape skins is not responsive to late RDI but can be down-regulated by early RDI, while *VvLAR2* transcript level is affected by neither of these two treatments (Castellarin et al., 2007a). With seasonal RDI, *VvLAR2* transcript abundance can be transiently elevated before veraison, and the similar response is also observed in the grape berries sprayed with ABA (Lacampagne et al., 2010; Villalobos-González et al., 2016; Caceres-Mella et al., 2017). Moreover, pre-veraison ABA application delayed the expression of *VvANR* and *VvLAR1*, which consistent with the lower *VvANR* and *VvLAR* enzymatic activities during PA biosynthesis active stage in grape berries (Lacampagne et al., 2010). Although exogenous ABA application to large extent resemble the PA gene expression patterns under water deficit, to how much extent ABA involves in the regulation of PA structural genes expression remains to be answered, and the missing components that disturb the effects of VvMYBPA1 on the expression PA structural genes under water deficit need to be further discovered.

Accompanied with the increased ABA concentration during pre-veraison under seasonal water deficit treatments, BR and GA_3_ levels are slightly decreased in grape berries in consecutive years (Yang et al., 2020b; Guo et al., 2021) (**Figure 2**). It has been shown that the spraying of BR before veraison enhanced the accumulation of anthocyanins and PAs in berry skins (Luan et al., 2013; Xi et al., 2013; Vergara et al., 2018), while exogenous application of GA_3_ does not significantly affect the levels of anthocyanins and PAs (Reynolds et al., 2016; Murcia et al., 2017; Kaplan et al., 2019; Gao et al., 2020; Tyagi et al., 2021), except for that the long-term 80 mg/L GA_3_ application on ‘Red globe’ promotes anthocyanin biosynthesis (Dong et al., 2023). The trade-off of ABA and BR levels has been proposed as the strategy to balance the stress response and development in plants (Wang et al., 2020b), which might also contribute to tuning the flux between PA and anthocyanin pathways under water deficit. In addition, ABA inhibits GA biosynthesis and induces GA degradation to increase drought tolerance of plants (Shohat et al., 2021; Mukherjee et al., 2023). Recently, VyMYB24 is found to confer the drought tolerance in Chinese wild *Vitis* species *V. yanshanesis* and negatively regulate GA_3_ level under drought stress (Zhu et al., 2022), and its homolog in *V. vinifera* is responsible for the enhanced flavone and terpenoid accumulation under drought and high light/UV conditions (Savoi et al., 2016; Lu et al., 2021; Zhang et al., 2023). Then further answering whether and how MYB24 participates in the interaction of phytohormone levels and flavonoid gene expression can help with understanding ABA-centered anthocyanins and PAs regulation network under water deficit (**Figure 2**).

## 3 The interaction of light and phytohormones in regulating the accumulation of flavonoid

**Figure 3:**
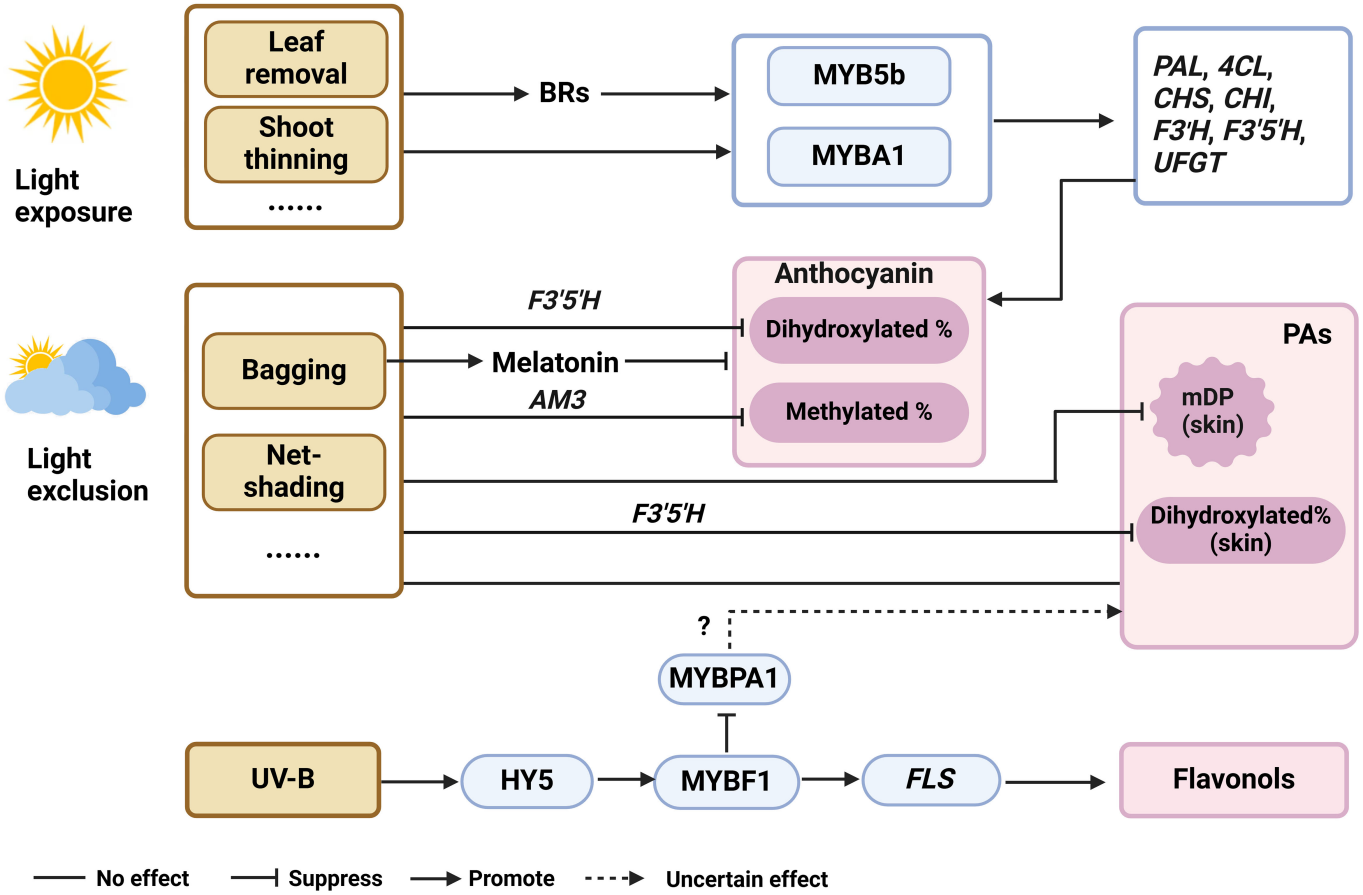
The roles of phytohormones in mediating the biosynthesis of flavonoid under various light conditions. mDP, mean polymerization degrees, BRs, brassinolide, ABA, abscisic acid, UV-B, ultraviolet-B, F3’5’H, flavanone 3’5’-hydroxylase, AM3, MATE3, PAL, phenylalanine ammonia-lyase; 4CL, 4-coumarate, CoA-ligase; CHS, chalcone synthase; CHI, chalcone isomerase; F3’H, flavanone 3’-hydroxylase; UFGT, UDP-glucose, flavonoid 3-*O*-glucosyltransferase; FLS, flavonol synthase.

In the viticulture practice, bagging, net-shading, and canopy managements (leaf removal and shoot thinning) are effective approaches to adjust both quantity and quality of sunlight around grape berry clusters. Exposure of grape berries to sunlight generally increases anthocyanin and flavonol levels in skins, mainly by activating the expression of phenylpropanoid genes (*VvPAL*, *Vv4CL*), flavonoid genes (*VvCHS*, *VvCHI*, *VvF3’H*, *VvF3’5’H*, *VvUFGT*), flavonol gene (*VvFLS*) and the relevant transcription regulators (*VvMYBA1, VvMYB5b, VvMYBF1 and VvHY5*) (Matsuyama et al., 2014; Guan et al., 2016; Czemmel et al., 2017; Li et al., 2023) (**Figure 3**). However, excessive sunlight exposure can result in the sunburn of berry clusters, which induces the degradation of anthocyanins, PAs and flavonols (Sun et al., 2017; Reshef et al., 2018; Torres et al., 2020). Flavonol biosynthesis is particularly sensitive to UV-B light and can be significantly inhibited in berries subjected to light exclusion using light-proof box (Koyama et al., 2012; Loyola et al., 2016). It was found that VvHY5 activated *VvMYBF1* transcript level under UV-B, which in turn regulated the expression of *VvFLS1*, *VvGT3* (encoding a glycosyltransferase) and *VvRHaT1* (encoding a rhamnosyltransferase), to promote flavonol accumulation. Additionally, VvMYBF1 negatively regulated the expression of *VvMYBPA1*, indicating a competition between the flavonol and PA branches (Czemmel et al., 2017). Under light exclusion, the proportion of dihydroxylated anthocyanins increased due to the down-regulation of *F3’5’H* expression (Downey et al., 2004; Azuma et al., 2012; Koyama et al., 2012; Matsuyama et al., 2014; Guan et al., 2016). Guan et al. (2016) also found the proportion of methylated anthocyanins increased. Light exposed and exclusion had little effect on PA content at harvest (Downey et al., 2004; Fujita et al., 2007; Koyama and Goto Yamamoto, 2008; Liu et al., 2015; VanderWeide et al., 2022) (**Figure 3**). This may be because the treatments are applied at the onset of veraison, while PAs are mainly synthesized in large quantities at the flowering stage. Shading grape clusters during the pre-veraison period, decreased PA levels in berry skins by down-regulating the expression of *VvLAR1* and *VvANR* before ripening (Downey et al., 2004; Jeong et al., 2004; Fujita et al., 2007; Koyama and Goto-Yamamoto, 2008; Liu et al., 2015) (**Figure 3**). Researchers speculate that extractable PAs decreased more markedly in sunlight-exposed conditions than in shaded berry skins during ripening, suggesting that light promotes the synthesis of insoluble PAs. Downey et al. (2004) and Fujita et al. (2007) also discovered there is no significant effect of light exclusion on the level of PAs in the seeds. While the proportion of the dihydroxylated subunits increased in berry skins, which agreed with the down-regulation of *VvF3’5’H* levels Koyama and Goto-Yamamoto, 2008). By Shading with light-proof boxes, this group further confirm that light exclusion decreased the mean degree of polymerization (mDP) of PAs in grape skin (Koyama et al., 2012).

Several studies have focused on endogenous hormones in grapes under light conditions. Guo et al. (2020) bagged two wine grape varieties, 'Cabernet Sauvignon' and 'Carignane', from fruit set until harvest and found the cluster bagging induced the melatonin synthesis. Meng et al. (2019) found the application of melatonin during the pre-veraison period at the concentration of 100 mg/L decreased the anthocyanin accumulation, while increasing the content of (+)-catechin and (-)-epicatechin in 'Merlot' berry skin. This suggests that light exclusion reduces anthocyanin accumulation by increasing melatonin levels (**Figure 3**). Previous research in our laboratory found that the transcriptional changes of genes required for the biosynthesis and signal transduction of auxin, ethylene, BR and ABA were in accordance with the flavonoid accumulation in light-exposed berries during development, indicating the importance of phytohormones on berry flavonoid biosynthesis in response to light (Sun et al., 2017). Guan et al. (2016) bagged the grapes from fruit set to maturity, and found that total anthocyanin levels were lower under light-exposed conditions, but ABA concentration was elevated at fruit setting stage, then the difference diminished. UV-B irradiation of grapes from pre-flowering until harvest had no effect on endogenous ABA levels (Berli et al., 2011), suggesting that ABA plays a limited role in mediating the light and UV-B effects on anthocyanin biosynthesis (**Figure 3**). Liu et al. (2016) analyzed endogenous ethylene biosynthetic and signal-transduction pathways under light and dark condition, and found no significant difference in the expression of genes involved during the grape development, suggesting that ethylene is not responsive to light. When grape clusters are sprayed with exogenous 24-epibrassinolide (a type of BR) under both light and dark conditions, the increase in anthocyanin levels is significantly greater under light condition. Correspondingly, the application of BR up-regulated the expression of flavonoid genes (*VvCHI1*, *VvCHS2*, *VvCHS3*, *VvDFR*, *VvANS*, *VvMYBA1*) under light conditions (Zhou et al., 2018) (**Figure 3**). Throughout grape development, light increases *VvBZR1* transcript, a key transcription factor positively regulating BR, suggesting that light likely increases endogenous BR content. Moreover, it has been repeatedly reported that exogenous supplement of BR enhances anthocyanin accumulation in grape skin (Luan et al., 2013; Xi et al., 2013; Vergara et al., 2018). These findings together indicate that BR and light have synergistic effects on anthocyanin biosynthesis in grapes. Additionally, BR signaling through BRI1-EMS-SUPPRESSOR 1 (BES1) typically inhibits flavonol synthesis to promote plant growth. However, under UV-B stress, this inhibition is lifted, leading to increased flavonol production in Arabidopsis (Liang et al., 2020). This BR-UV-flavonoid interaction network may also help with understanding the flux control between flavonols and anthocyanins/PAs, but it remains to be testified in grapevine in further studies.

## 4 The limited understanding of phytohormones in mediating the response of the flavonoid pathway to temperature changes

**Figure 4:**
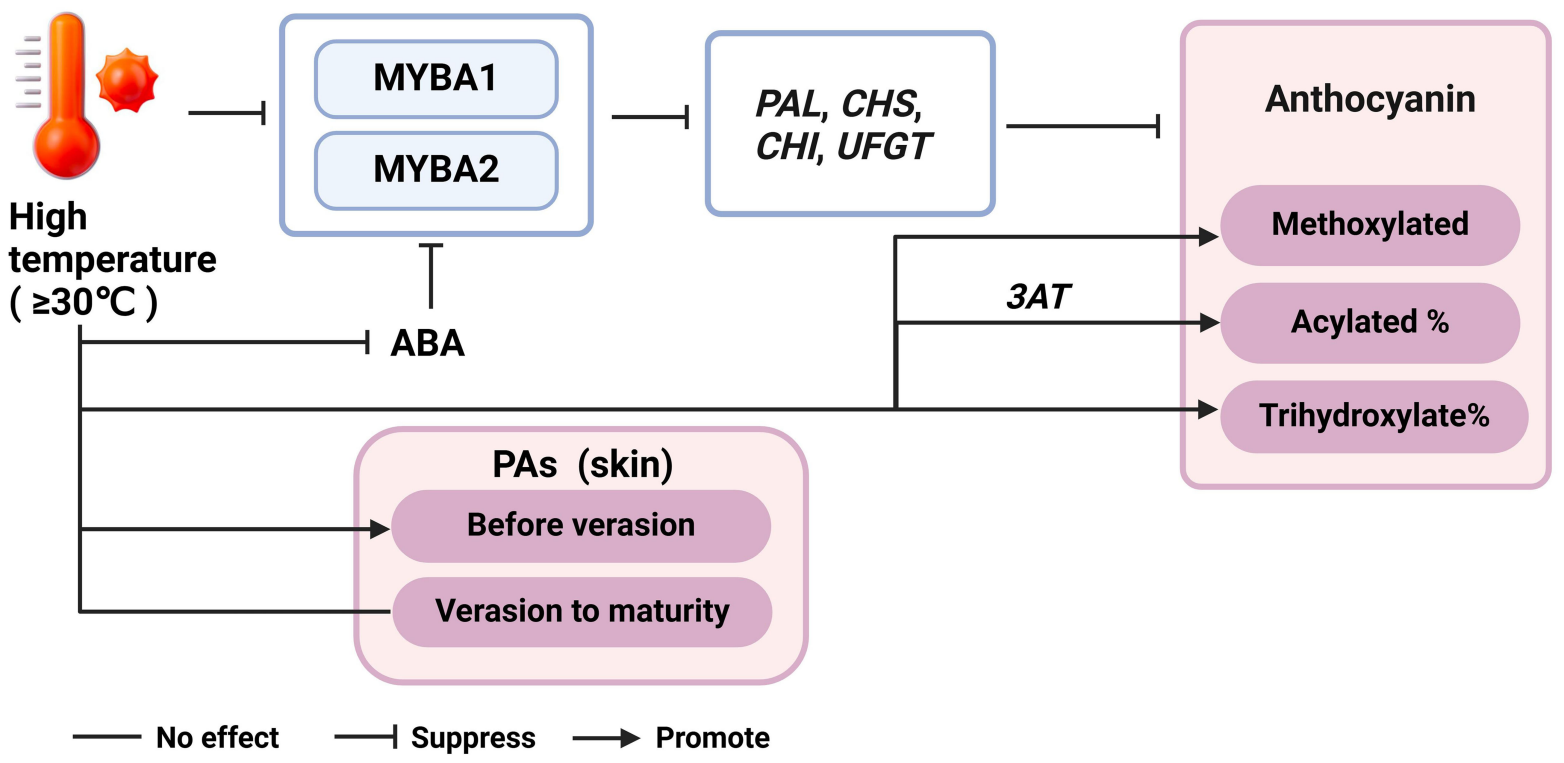
The regulatory network of temperature, phytohormones and flavonoid accumulation. ABA, abscisic acid; PAL, phenylalanine ammonia-lyase; CHS, chalcone synthase; CHI, chalcone isomerase; UFGT, UDP-glucose, flavonoid 3-*O*-glucosyltransferase.

Climate models predict an increase in both average and extreme atmospheric temperatures (Santos et al., 2020; Gashu et al., 2023). The effect of elevated temperatures on flavonoids metabolism has been a focal point in grape research area. Most studies have demonstrated the inhibitory impact of high temperature (equal to or above 30°C) on anthocyanin. Molecular analysis has revealed decreased transcription of phenylpropanoid and flavonoid structural genes, including *VvPAL*, *VvCHS*, *VvCHI*, and *VvUFGT*, as well as transcription factor genes such as *VvMYBA1* and *VvMYBA2* under high temperature (Yamane et al., 2006; Mori et al., 2007b; Lecourieux et al., 2017; Pastore et al., 2017; Yan et al., 2020) (**Figure 4**). In addition to repressing anthocyanin-related genes, high temperatures have been shown to increase peroxidase activity in cells, promoting anthocyanin degradation (Mori et al., 2007b; Yan et al., 2020). High temperatures also alter anthocyanin composition, increasing the proportion of acylated and tri-hydroxylated anthocyanins, which is accompanied by enhanced expression of *Vv3AT* (Lecourieux et al., 2017; Pastore et al., 2017; Yan et al., 2020). Elevated temperatures reduce endogenous ABA levels, as well as the expression levels of *VvMYBA1* and most flavonoid structural genes, suggesting that ABA may play a role in mediating the response of the flavonoid pathway to temperature changes (Yamane et al., 2006; Koshita et al., 2007; Azuma et al., 2012) (**Figure 4**). Mori et al. (2005a); Mori et al. (2005b) demonstrated that application of 250 ppm ABA through spraying almost diminished the adverse impact of night-time high temperature treatment on the reduction of anthocyanin levels in grape berries. Furthermore, Mori et al. (2005a) reported that high night temperatures (30°C) from veraison to maturity, compared to low night temperatures (15°C), reduced anthocyanin concentration in grape berry skins by inhibiting the expression of *VvCHS*, *VvF3H*, *VvDFR*, and *VvUFGT*, while flavonol levels remained unchanged. Gaiotti et al. (2018) also found low night temperature (10°C) during veraison favors the accumulation of anthocyanin by upregulating *VvCHS*, *VvF3H*, *VvUFGT* and *VvMYBA1*. Furthermore, Yan et al. (2020) observed that day temperature exerts a stronger influence on anthocyanin accumulation than night temperature. Their study, which involved setting different day and night temperature regimes, revealed that higher day temperatures led to a more pronounced decrease in anthocyanin concentration compared to variations in night temperatures.

The effect of temperature on PAs and flavonols accumulation is still not well understood. High night temperatures (above 8°C compared to environmental temperatures) during PA biosynthesis stages promoted flavan-3-ols synthesis in grape skins, while these differences were no longer significant after veraison until maturity, and no effect on flavonols regardless of the period of treatment (Cohen et al., 2012a; Cohen et al., 2012b). Pastore et al. (2017) found that PAs were not affected by heat stress (average temperature 26°C), while flavonols were decreased in Sangiovese grape skins. Similarly, studies by Gouot et al. (2019b) and Wu et al. (2019) observed no significant effect on flavan-3-ol monomer and PA levels in grape skins and seeds. In the study by Wu et al. (2019), a moderate increase in grape temperature was achieved by creating a local greenhouse effect, which led to an increase in PA levels before veraison but a decrease thereafter (**Figure 4**). This indicates that the timing and duration of heat exposure (at least 1.5°C higher than the ambient temperature) are crucial in determining the impact on PA accumulation. Overall, these findings highlight the nuanced and complex nature of temperature effects on grape phenolic compounds, emphasizing the need for further research to fully understand these interactions and their implications for viticulture under changing climatic conditions (Cohen et al., 2012a; Cohen et al., 2012b; Pastore et al., 2017; Gouot et al., 2019b; Wu et al., 2019).

## 5 Conclusion and prospect

In general, flavonoid metabolism constitute a complex process in grape. Environmental factors including water, light and temperature, significantly affect their biosynthesis. It is worth noting that there are interactions between these factors in field practice. For instance, bagging not only affects the light intensity, but also impacts on the temperature of grape. Therefore, the investigation of the interaction of various environmental factors on flavonoid metabolism is crucial for future research. Moreover, PAs is mainly synthesized at the flowering stage, so treatment of environmental factors at flowering stage may be more significant for the study of PAs regulation mechanism. Phytohormones signal network is complex and interactive during grape development. They play an important role as a medium in the process of studying environmental regulation of flavonoid. But there are limited studies that have conducted in-depth research, especially for temperature and light.

Additionally, although gene editing technology has been applied to optimize crop traits in other species such as rice and tomato, its implementation in grapes remains challenging due to the inhibition of polyphenols and other related antioxidant pathways (Osakabe et al., 2018). Consequently, scientists are focusing on more traditional hybridization methods to achieve the desired flavonoid characteristics in grapes (Yu et al., 2019). In the short term, adjusting the microclimate (water, light, and temperature) and hormone levels in grapes may be the most valuable strategy in the field. Furthermore, these cultivation treatments can help identify genes related to environmental factors and plant hormones, which can serve as breeding markers for regulating flavonoid traits.
